# A Norbornene-Derived Epoxy/Cyanate Ester System with Enhanced Thermal and Dielectric Properties as Electronic Materials

**DOI:** 10.3390/molecules31162800

**Published:** 2026-08-11

**Authors:** Peng Zhao, Jianming Zhang, Li Li, Long Zhao

**Affiliations:** Institution of Quantum and Sustainable Technology (IQST), School of Chemistry and Chemical Engineering, Jiangsu University, 301 Xuefu Road, Zhenjiang 212013, China; 2222312043@stmail.ujs.edu.cn (P.Z.); zhangjm@ujs.edu.cn (J.Z.)

**Keywords:** epoxy, norbornene, dielectric property, thermal stability, electronic material

## Abstract

In this work, to meet the requirements of high-performance electronic devices for high-frequency/speed telecommunication and semiconductor packaging, we developed a norbornene (NB)-backboned epoxy/cyanate ester compound system to modify the commercial bisphenol-A (BPA) diglycidyl ether (DGEBA) epoxy resin. A norbornene-based epoxy monomer (ENBDE) and cyanate ester (ENBCY) were synthesized using 5-ethylidene-2-norbornene (ENB) as the key starting material. The ENBDE was blended with DGEBA as the epoxy resin compound, which was cured using ENBCY as the curing agent to form a cross-linked thermoset. The effect of ENBDE content on thermal stability, mechanical properties, dielectric performance, and bonding strength of the thermosets was investigated. The optimized formula showed a significantly improved thermal stability of the cured resin with a glass-transition temperature of 249.2 °C and a 5% weight-loss temperature (T_5%_) of 351.5 °C; the dielectric constant and dissipation factor at a high frequency of 10 GHz were measured to be as small as 2.57 and 0.0068, respectively, showcasing great potential as a high-performance dielectric epoxy material for high-frequency/speed electronic applications.

## 1. Introduction

The rapid evolution of high-speed, high-integrity electronic systems—ranging from radio-frequency (RF) modules to advanced semiconductor packaging for Artificial Intelligence (AI) and High-Performance Computing (HPC)—has imposed rigorous demands on material performance [1,2]. Increasing integrated circuit (IC) densities and the pursuit of minimal propagation delays necessitate advanced electronic materials capable of withstanding elevated operating temperatures while maintaining long-term reliability. Within this landscape, epoxy resins are indispensable. These epoxy thermosetting polymers, such as the standard diglycidyl ether of bisphenol-A (DGEBA)-based epoxies, are widely utilized in printed circuit boards (PCBs), electronic component encapsulation, and structural adhesives due to their robust mechanical integrity, superior adhesion, and excellent electrical insulation [2,3,4].

In the regime of high-frequency microelectronics, the thermal and dielectric performance of a substrate is the primary determinant of device longevity and signal fidelity. The glass transition temperature (T_g_) serves as the critical threshold for mechanical reliability; it represents the point where the polymer transitions from a rigid, glassy state to a compliant, rubbery state. For high-density interconnects (HDIs) and PCBs, maintaining a high T_g_ is essential to prevent dimensional instability and internal stress during solder reflow or high-power operations. When a material operates near its T_g_, the resulting thermal expansion can lead to micro-cracking at the copper–epoxy interface, eventually causing catastrophic circuit failure. From an electromagnetic perspective, the dielectric constant (*D_k_* or relative permittivity) and the dissipation factor (*D_f_* or loss tangent) dictate the efficiency of signal propagation. According to the relationship:

*V*_p_ = *C/D_k_*^1/2^ (1)

where *V*_p_ is the signal velocity and *C* is the speed of light. In AI and HPC architectures, where nanosecond delays are critical, reducing *D_k_* is the most effective way to minimize propagation delay. Similarly, the signal-transmission loss (*L*) varies with *D_k_* and dissipation factor (*D_f_*) as follows:

*L* = *K* × *(f/C)* × *D_f_* × *D_k_*^1/2^ (2)

where *K* is a dimensionless proportionality constant dependent on material composition, molecular structure and test environment, and *f* stands for the operating frequency. Clearly, a lower *D_k_* and *D_f_* favor faster signal transmission. More importantly, the *D_f_* quantifies the electrical energy converted into thermal energy within the dielectric. As frequencies move into the 10 GHz and millimeter-wave ranges, the molecular dipoles within the polymer (such as residual hydroxyl groups in standard epoxies) struggle to keep pace with the rapidly alternating electric field. This friction results in dielectric heating and signal attenuation. Consequently, achieving ultralow-loss performance requires a molecular architecture that minimizes polar groups and restricts the rotational freedom of the polymer segments, ensuring that signal intensity remains high over long transmission distances [4,5].

Despite their ubiquity, commercial DGEBA epoxies exhibit inherent limitations [6], such as moderate thermal stability (Tg < 120 °C) and relatively high dielectric loss (*D_k_* > 3.0 and *D_f_* > 0.03) at >5 GHz frequencies [7]. Consequently, there is a burgeoning interest in alternative molecular architectures. Norbornene (NB) and its derivatives, such as 5-ethylidene-2-norbornene (ENB), offer a rigid, hydrophobic, and saturated bicyclic framework [8,9,10,11,12,13]. Integrating NB moieties into a polymer network can effectively lower dipole density and restrict segmental chain motion while increasing fractional free volume—attributes that collectively enhance thermal stability and diminish dielectric loss [5,10]. These monomers exhibit good compatibility with a range of thermosetting resin systems, enabling flexible formulation design. In addition, they are widely accessible as by-products of large-scale petrochemical processes, providing notable cost efficiency and practical advantages for industrial production [14,15]. Furthermore, replacing traditional polyamine hardeners with cyanate ester resins can further optimize performance, as cyanate esters are noted for their exceptional thermal resistance and dielectric compatibility with epoxy systems [16].

In this study, using ENB as the key material, we synthesized two NB-based resins of ENB-epoxy (ENBDE) and cyanate (ENBCY). The ENBDE was blended with commercial DGEBA at different ratios to form epoxy compound, and cured using ENBCY as the curing agent. Thermal, mechanical, and dielectric characterizations revealed that the addition of ENBDE into a DGEBA-based epoxy cured with ENBCY significantly enhances thermal and mechanical properties of the thermosets, achieving a T_g_ as high as 249.2 °C and a 5% weight-loss temperature of 351.5 °C. Furthermore, the cured resins exhibit ultralow *D_k_* and *D_f_* of 2.57 and 0.0068 at a high frequency of 10 GHz, respectively, attributed to the introduction of NB units to the polymer frameworks and the incorporation of ENBCY. This work introduces a promising approach to improve the performance of conventional epoxy resins for high-frequency electronic applications.

## 2. Results and Discussion

**Figure 1:**
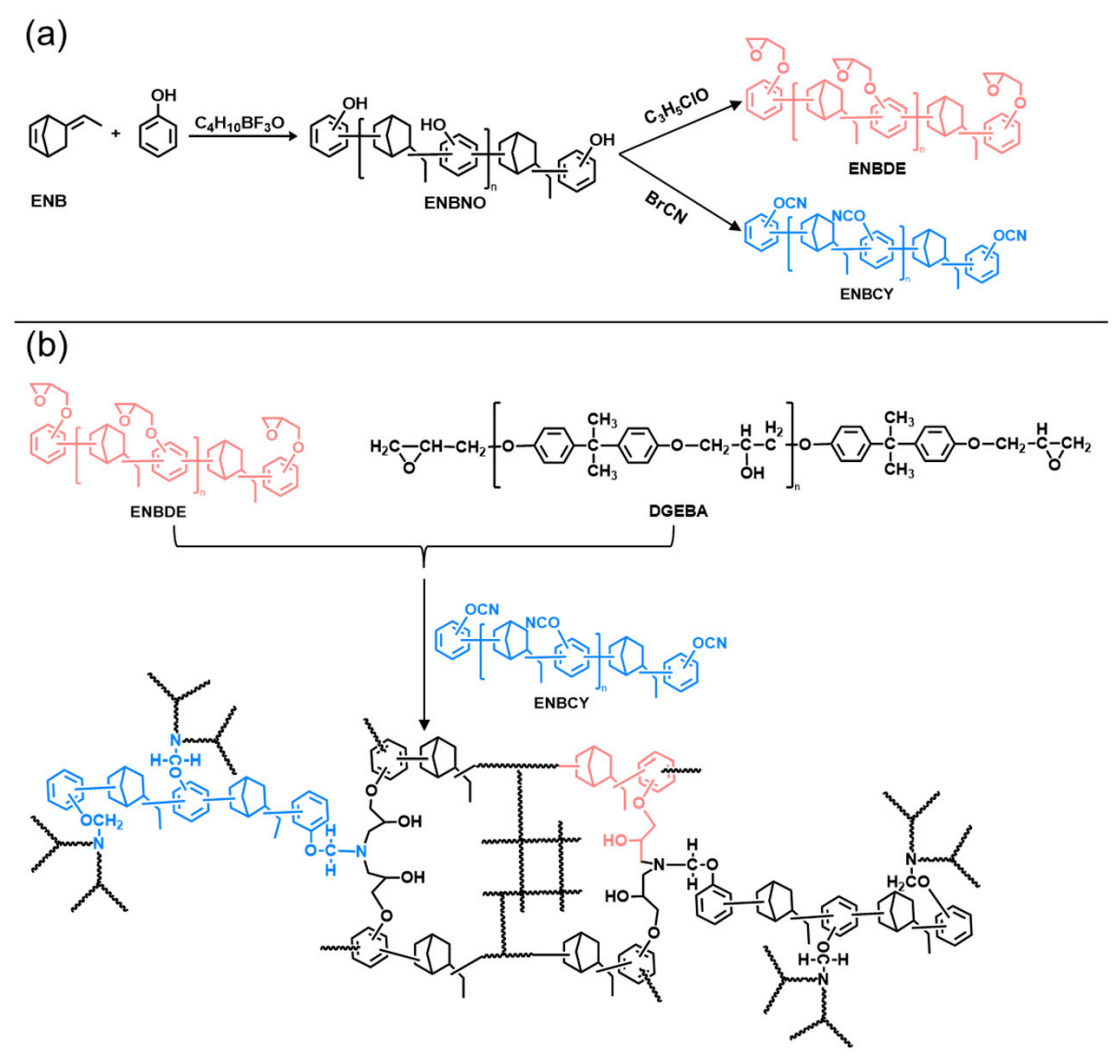
Schematic illustration for the synthesis of the ENBDE and ENBCY (**a**) and the crosslink of ENBDE/DGEBA compound using ENBCY (**b**).

As depicted in Figure 1a, the synthesis of the NB-based epoxy ENBDE follows an optimized two-step process, closely resembling the industrial-scale production of epoxy resins. In the first step, phenol undergoes a classic Friedel–Crafts alkylation with ENB in the presence of boron trifluoride etherate as a catalyst, yielding the phenol-ENB intermediate (ENBNO) [5]. In the second step, under alkaline conditions, ENBNO reacts with epichlorohydrin in an epoxidation reaction, which is consistent with the standard commercial epoxy resin production process, leading to the formation of ENB-phenol diglycidyl ether, ENBDE. The gel permeation chromatography (GPC) results show that the number-average molecular weight (M_n_) of ENBDE is approximately 1620, the weight-average molecular weight (M_w_) is measured to be around 1880, and the polydispersity index is ~1.16, classifying it as a medium-low oligomer, ideal for constructing a crosslinked network. Furthermore, the NB-based cyanate ester was synthesized via a similar two-step process, in which ENBNO undergoes nucleophilic substitution with bromine cyanide (BrCN) to form ENBCY with a conversion ratio estimated to be ~93%.

**Figure 2:**
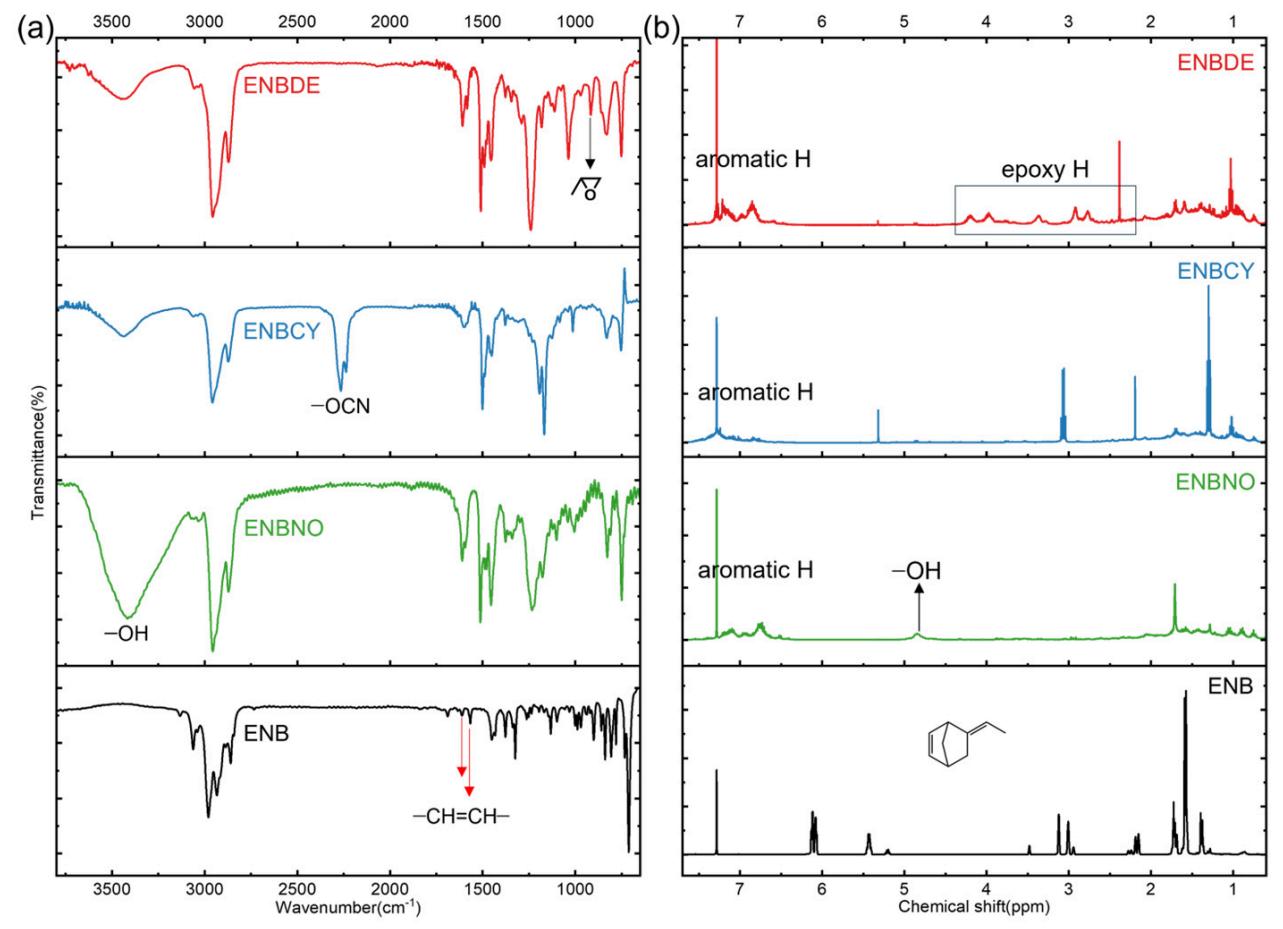
IR (**a**) and ^1^H-NMR (**b**) spectra of ENB, ENBNO, ENBCY and ENBDE.

The chemical structures of ENB, ENBNO, and the final products ENBDE and ENBCY were characterized using Infrared spectroscopy (IR) and proton nuclear magnetic resonance (^1^H-NMR). As shown in the IR spectrum of ENB (Figure 2a), the characteristic absorption peak of the –HC=CH– in the ENB molecule disappears completely after reacting with phenol, indicating the consumption of the double bond. ENBNO shows new absorption peaks between 1450 cm^−1^ and 1490 cm^−1^, which correspond to the aromatic ring vibrations, along with a broad absorption band between 3066 cm^−1^ and 3755 cm^−1^, attributed to the stretching vibration of phenolic hydroxyl groups. After the epoxidation reaction, a new characteristic peak appears around 910 cm^−1^, corresponding to the deformation vibration of the epoxy ring [17]. Following nucleophilic substitution with bromine cyanide, a new feature peak is observed around 2250 cm^−1^ in ENBCY, and the hydroxyl peak weakens.

The ^1^H-NMR spectra shown in Figure 2b further validate the structural evolution during the stepwise reactions. The ENB group displays typical aliphatic proton signals, with characteristic olefinic proton peaks appearing at 5.4 ppm and 6.1 ppm corresponding to the resonance of –CH=CH–. In comparison, the olefinic proton signals completely disappear in the ENBNO spectrum, indicating full consumption of the double bonds during the phenol-ENB reaction. The aromatic proton signals from the phenolic unit appear as expected, consistent with the theoretical structure. For ENBDE and ENBCY, the hydroxyl peaks are both significantly weakened, during the synthetic process, while new proton resonances of epoxy groups appear in ENBDE at 2 ppm–4.5 ppm. Both IR and ^1^H-NMR characterization results collectively confirm the successful synthesis of the NB-based epoxy and cyanate ester resins.

**Figure 3:**
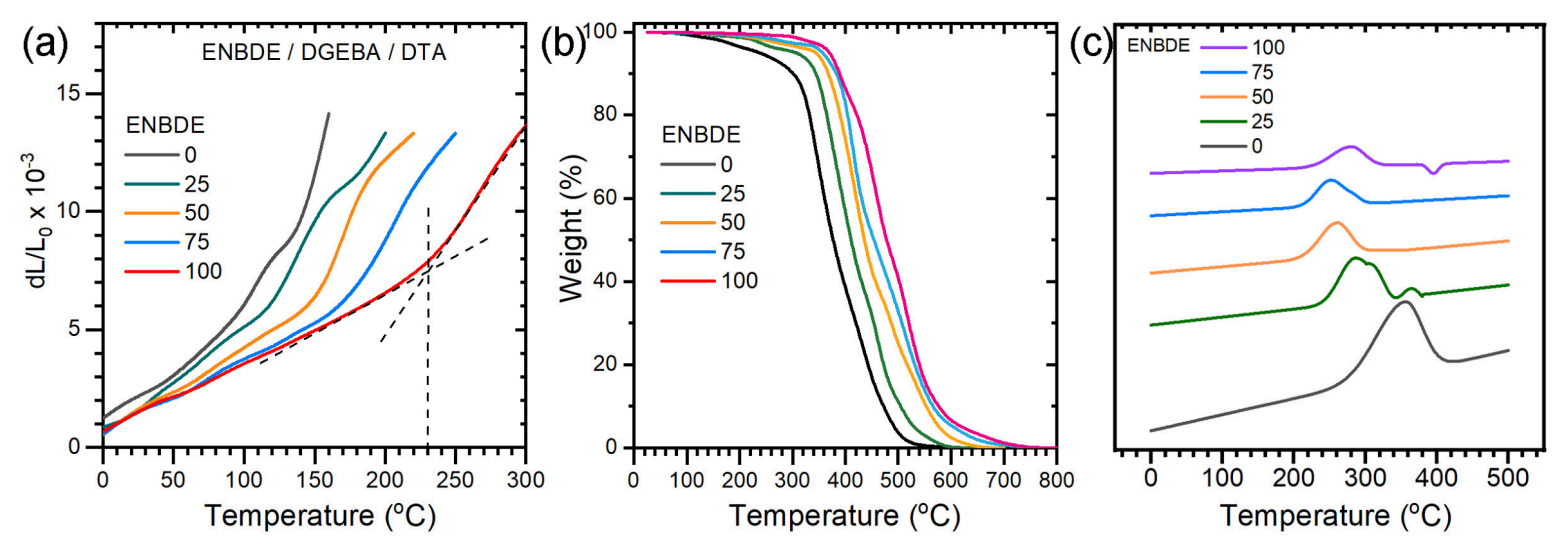
(**a**) TMA (the vertical axis represents relative deformation (dL/L_0_), referring to the ratio of dimensional change to the original sample length), (**b**) TGA and (**c**) DSC curves of the epoxy resin system with different ENBDE content.

Thermoset samples were prepared by blending ENBDE with commercial DGEBA at various mass ratios, followed by mixing with 20 wt% of ENBCY as curing agent to form a three-dimensional (3D) crosslinked network of ENBDE/DGEBA/ENBCY upon heating. The thermal properties of the resulting thermosets were evaluated using thermogravimetric analysis/Differential Scanning Calorimetry (TGA/DSC) and thermomechanical analysis (TMA), with performance compared to the solely DGEBA system (ENBDE:DGEBA = 0:100). The corresponding TMA and TGA results are shown in Figure 3a and Figure 3b, respectively. A clear, monotonic increase in both the T_g_ and the 5% weight-loss temperature (T_5%_) was observed as the ENBDE content increased. As summarized in Table 1, the fully NB unit backboned thermoset, i.e., ENBDE:DGEBA = 100:0, exhibited a T_g_ of 249.2 °C and a T_5%_ of 351.5 °C, significantly higher than those of the unmodified DGEBA control. Even formulations with relatively low ENBDE content (e.g., ENBDE:DGEBA = 25:75) showed enhanced thermal stability compared to the neat DGEBA system, highlighting the substantial contribution of the rigid NB skeleton to the network structural integrity. These findings collectively demonstrate that incorporating NB-based units effectively enhances the thermal robustness of the epoxy/cyanate ester hybrid networks, resulting in materials with significantly better thermal resistance.

The marked increase in T_g_, from 95.4 °C in the neat DGEBA resin system to 249.2 °C of the pure ENBDE one, reflects a significant change in the thermomechanical properties of the polymer network. In principle, an elevated T_g_ in thermosets generally signifies an increased crosslink density and a more compact, rigid network structure [18,19]. The NB backbone, with its rigid, saturated bicyclic structure, acts as a high-impedance anchor within the crosslinked network. These NB units notably raise the energy barrier for polymer chain movement, effectively locking the 3D network structure and requiring substantially more thermal energy to initiate the glass-to-rubber transition. The TGA data, showing a T_5%_ increase from 247.6 °C to 351.5 °C, further illustrates the superior thermal stability of the NB-phenol linkages. In contrast to the relatively unstable isopropylidene bridge in DGEBA, the saturated bicyclic structure of ENBDE is much less prone to early thermal scission. This enhanced structural integrity, combined with the high crosslink density introduced by the ENBCY curing agent, ensures that the network retains its mechanical strength even at elevated temperatures, where traditional epoxies would degrade significantly.

DSC was further employed to investigate the curing behavior of the blended systems. As displayed in Figure 3c, all samples exhibited a single exothermic curing peak, demonstrating that epoxy resins (ENBDE/DGEBA) underwent homogeneous co-curing with the cyanate ester (ENBCY), and no phase separation occurred in these systems. The curing peak temperature gradually decreased as the ENBDE content increased from 0 to 75%, with the neat DGEBA system (ENBDE:DGEBA = 0:100) presenting a curing peak at around 330 °C. By contrast, a slight elevation in curing peak temperature was observed when the ENBDE content was further increased to 100%. This phenomenon can be explained as follows: within an appropriate composition range, the rigid NB skeleton of ENBDE improves the reactivity of epoxy groups. It accelerates ring-opening polymerization and promotes the formation of triazine networks through reactions with ENBCY, thus reducing the curing temperature. Nevertheless, the densely packed rigid bicyclic structures in the neat ENBDE system generate prominent steric hindrance, which restricts molecular chain mobility and hinders the collision of functional groups. Consequently, a higher temperature is required to complete the curing reaction. In addition, all formulations showed comparable exothermic peak intensities, verifying that full crosslinking was accomplished for all samples.

**Figure 4:**
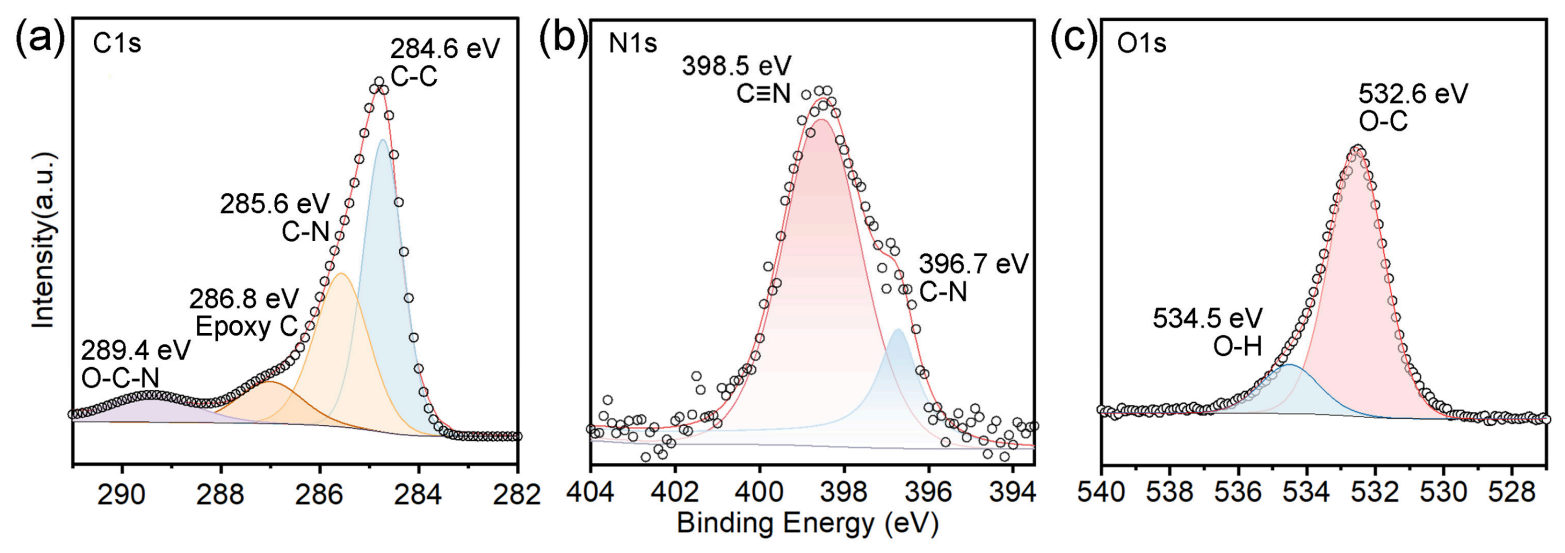
XPS spectra of the C 1s (**a**), N 1s (**b**) and (**c**) O 1s for the cured resin of ENBDE:DGEBA:ENBCY = 0:100:20.

X-ray photoelectron spectroscopy (XPS) was used to characterize the cured resin (ENBDE:DGEBA:ENBCY = 0:100:20) and analyze its chemical composition. As illustrated in Figure 4a, in the C 1s spectrum, the peak at 284.6 eV can be assigned to the C–C/C–H skeletal carbon, and the peak at 285.6 eV corresponds to the C–N carbon, due to the use of ENBCY; the peak at 286.8 eV can be attributed to carbon atoms of epoxy groups and C–O bonds formed after epoxy ring opening; the band at 289.4 eV corresponds to the carbon of the O–C–N structure [20,21]. In the N 1s spectrum (Figure 4b), the peak at 398.5 eV is highly likely to be the residual C≡N structure of isocyanate, and the peak at 396.7 eV corresponds to the C–N structure formed via cross-linking between epoxy and isocyanate groups. In the O 1s spectrum (Figure 4c), the peak at 532.6 eV belongs to C–O bonds of ether and ester groups, and the peak at 534.5 eV can be assigned to hydroxyl groups produced by epoxy ring opening [20,21]. These results verify that isocyanate as an effective curing agent reacts with the epoxy matrix during curing to form a cross-linked network, and 20 wt% of ENBCY is sufficient to cure the resin.

**Figure 5:**
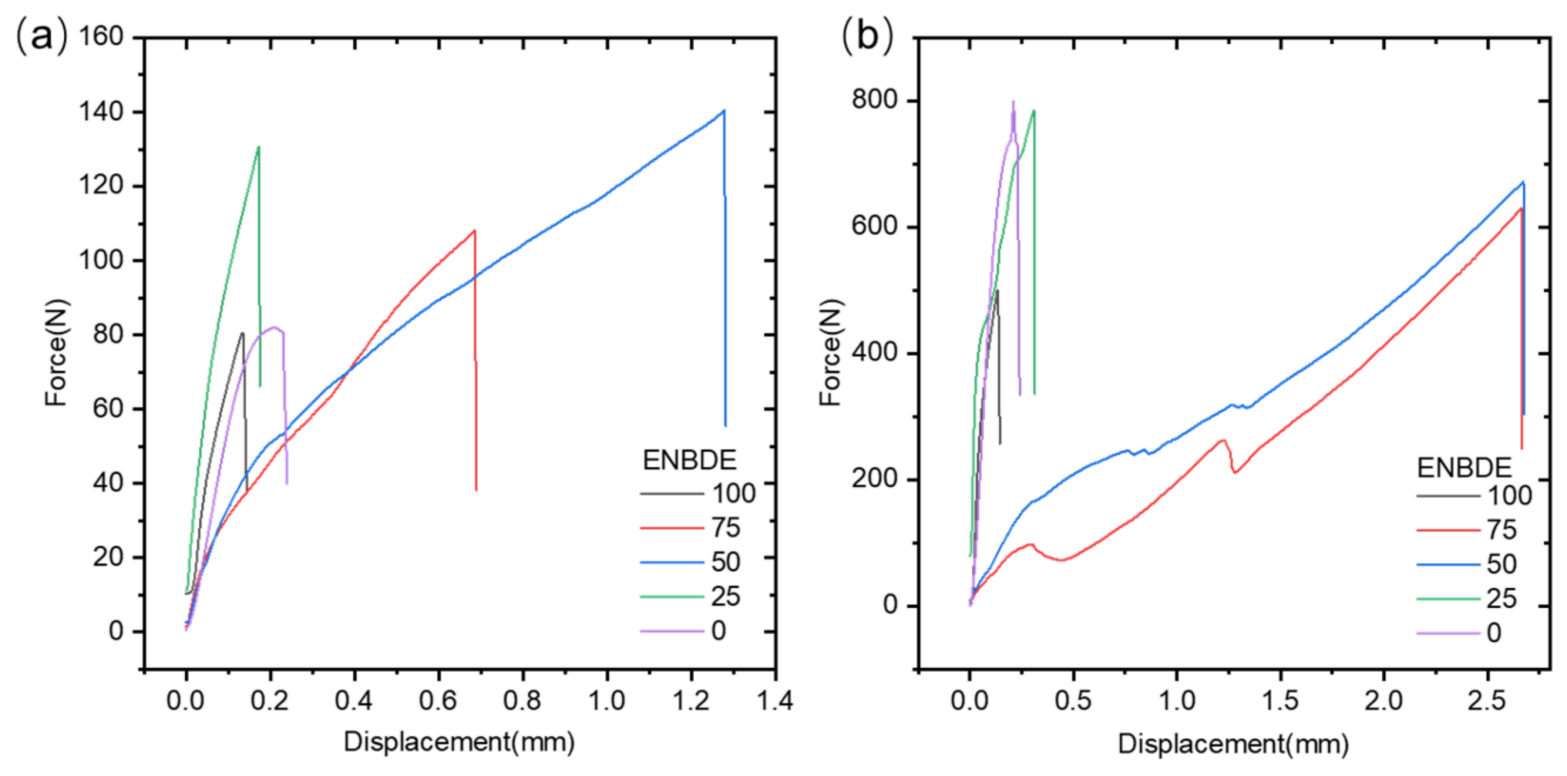
Stress–strain curve for tear strength (**a**) and tensile strength (**b**).

The mechanical properties of the cured epoxy systems, including tensile strength and tear resistance, were evaluated to clarify the influence of ENBDE incorporation on the structural integrity of crosslinked networks. Figure 5 shows the stress–strain curves of the materials under tensile loading. All samples exhibit typical tensile characteristics of polymer materials. In the initial stage, the curves present a monotonically increasing characteristic, indicating that the materials undergo elastic deformation. With the increase in strain, the materials gradually enter the plastic deformation stage until fracture. Combined with the thickness and width of test specimens, the tensile strength and tear strength listed in Table 2 were obtained from the data derived from Figure 5. Notably, samples with either low or excessively high ENBDE content show obvious brittle behavior. They fracture rapidly after reaching the maximum stress with small strain at break. By contrast, specimens with moderate ENBDE content possess larger elongation at break and better plastic deformation capacity, demonstrating improved ductility and toughness. It can be concluded that a moderate dosage of ENBDE is beneficial to optimize the comprehensive mechanical performance of the epoxy network.

As summarized in Table 2, the tensile strength increases first and then decreases with the rising proportion of ENBDE in the ENBDE:DGEBA blends. The maximum tensile strength of 43.14 MPa is achieved at an ENBDE:DGEBA mass ratio of 25:75. Neat DGEBA (ENBDE:DGEBA = 0:100) shows a tensile strength of 40.64 MPa, while the value gradually drops to 27.32 MPa for pure ENBDE (ENBDE:DGEBA = 100:0). The tear strength exhibits a similar trend and reaches its peak at an ENBDE:DGEBA mass ratio of 50:50. Similarly, Shore D hardness increases initially and then declines with increasing ENBDE content, attaining the optimal value of 82.6 at an ENBDE:DGEBA mass ratio of 25:75. The rigid norbornene (NB) skeleton effectively improves the rigidity and T_g_ of the crosslinked epoxy networks.

These results reveal a distinct trade-off between network rigidity, T_g_ and tensile strength. The rigid bicyclic NB moieties increase the crosslink density and enhance the overall stiffness and thermal resistance of cured resins. Nevertheless, excessive incorporation of ENBDE leads to overly rigid network structures, which restrict molecular chain mobility and hinder stress transfer during stretching, thus reducing the tensile strength. Overall, the introduction of NB-based units enables the modulation of comprehensive properties of epoxy materials. A moderate dosage of ENBDE is indispensable to balance mechanical strength, structural rigidity and thermal stability.

**Figure 6:**
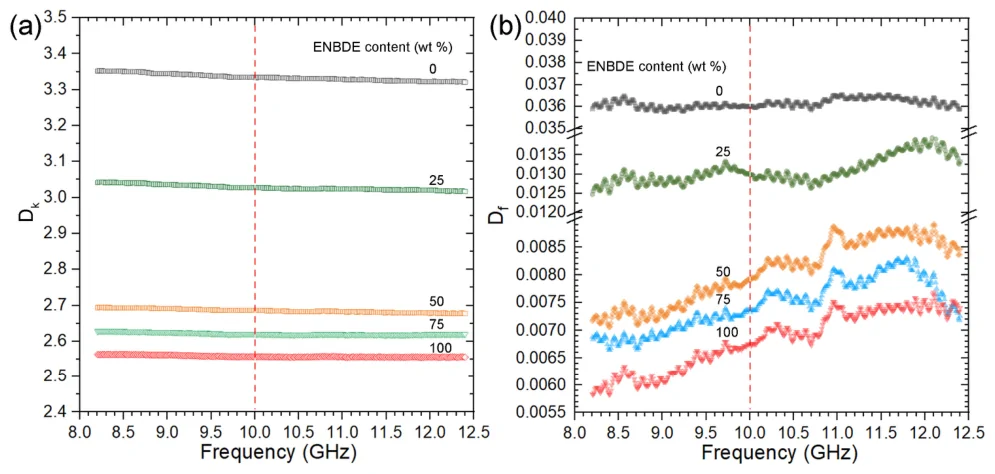
Dielectric properties of *D_k_* (**a**) and *D_f_* (**b**) tested in the range of 8 to 12 GHz.

To meet the rigorous demands of high-frequency/high-speed electronic application, such as chip packaging, the dielectric performance, moisture resistance and bonding strength of the cured ENBDE/DGEBA/ENBCY systems were systematically evaluated. As presented in Figure 6 and Table 3, the results reveal a clear, favorable trend: as the ENBDE content increases, both *D_k_* and *D_f_* decrease significantly from *D_k_* > 3.0 and *D_f_* > 0.03 for ENBDE:DGEBA = 0:100 to *D_k_* < 2.6 and *D_f_* < 0.0075 for ENBDE:DGEBA = 100:0, in a high-frequency range from 8 GHz to 12 GHz. Notably, at 10 GHz of a critical industry standard, the pure ENBDE-based formulation, i.e., ENBDE:DGEBA = 100:0, exhibits an ultra-low *D_k_* of 2.57 and *D_f_* of 0.0068, representing a dramatic reduction from the values of the unmodified DGEBA control (*D_k_* = 3.33, *D_f_* = 0.036). Even moderate ENBDE loadings (e.g., 50 wt%) yield substantial improvements, with *D_k_* and *D_f_* dropping to 2.69 and 0.0079, respectively, demonstrating the efficacy of the NB structure in tailoring the dielectric response.

This remarkable dielectric performance improvement arises from a combination of structural effects introduced by the ENBDE component. First, the non-polar, saturated NB bicyclic backbone effectively dilutes the density of polar functional groups (such as hydroxyl and ether linkages) within the crosslinked network, reducing overall molecular polarizability. Second, the bulky, rigid NB units introduce significant steric hindrance, which restricts the rotation and reorientation of remaining polar dipoles under high-frequency alternating fields, thereby minimizing energy dissipation. Additionally, the kinked, non-planar geometry of the NB structure disrupts efficient chain packing, increasing the fractional free volume of the polymer matrix. Since air has a much lower dielectric constant than polymer resins, this increased free volume further lowers the overall *D_k_* of the material. Collectively, these effects address the primary mechanisms of dielectric loss in conventional epoxies, resulting in the exceptional high-frequency performance observed here.

The moisture absorption behavior, which is critical for long-term dielectric stability, also shows a strong dependence on ENBDE content. As shown in Table 3, the water uptake decreases progressively from 2.42% for neat DGEBA to as low as 0.68% for the pure ENBDE system [8,10]. This improvement is attributed to the inherently hydrophobic nature of the aliphatic NB framework, which reduces the formation of hydrogen-bonding sites and limits water penetration into the polymer matrix. By suppressing moisture ingress, the ENBDE-modified systems avoid the significant increase in *D_k_* and *D_f_* typically caused by absorbed water, ensuring consistent dielectric performance under humid operating conditions. Furthermore, the copper bonding strength of all formulations remains stable around 0.20 kN–0.29 kN, comparable to the neat DGEBA system, indicating that the introduction of ENBDE and ENBCY does not compromise the interfacial adhesion essential for practical electronic applications.

Furthermore, the dielectric properties of the as-prepared resin system were compared with those of epoxy matrices reported in recent literature. As summarized in Table 4, while many recently reported epoxy systems have only been evaluated at lower frequencies (e.g., 1 MHz or 1 GHz), the dielectric properties in this work were rigorously evaluated at a high frequency of 10 GHz. Driven by rapid technical innovation in the electronics market, testing at 10 GHz has emerged as a critical benchmark and industry standard for validating substrates intended for high-frequency/high-speed PCBs and RF chip packaging. Particularly in East Asia, the global hub for advanced electronic manufacturing, 1 GHz–5 GHz testing is no longer sufficient to meet the strict qualification standards for high-tier electronic materials. Because *D_f_* typically intensifies at elevated frequencies due to dipole orientation limits, demonstrating an ultra-low *D_k_* of 2.57 and *D_f_* of 0.0068 under 10 GHz conditions highlights the profound practical readiness of this NB-derived system for next-generation telecommunication applications. Consequently, the present resin system exhibits superior dielectric performance relative to most epoxy-based materials documented in previous studies.

Beyond intrinsic molecular modification, a common industrial alternative involves the incorporation of micro- or nano-scale inorganic fillers into polymer matrices. For instance, the addition of specialized ceramic or nitride particles has been widely utilized to regulate the *D_k_* and *D_f_* over varying operating frequencies and temperatures by manipulating interfacial polarization and percolation thresholds [33]. However, high filler loading can introduce structural defects, air voids, and localized interfacial relaxation phenomena that complicate high-frequency signal integrity. In this work, we focus instead on a pure, intrinsic polymer structure engineering strategy to realize competitive low-*D_k_* and low-*D_f_* values without relying on auxiliary inorganic fillers. This ensures a macroscopically homogeneous material with minimal polarization degradation at high frequencies. Future developments building upon this NB-derived epoxy/cyanate ester matrix will explore the synergistic incorporation of targeted low-dielectric fillers to further expand its thermodielectric operational envelope in next-generation high-speed electronic packaging.

## 3. Materials and Methods

### 3.1. Characterization

IR spectra were recorded using a Thermo Fisher Nicolet iS50 spectrometer (Waltham, MA, USA). The ^1^H-NMR spectra were acquired on a Bruker Avance II 400 MHz instrument (Billerica, MA, USA). Gel permeation chromatography (GPC) was performed using a Shimadzu LC-20A to determine molecular weights (Nakagyo-ku, Japan). XPS measurements were performed on a Thermo Scientific Escalab QXi spectrometer (Waltham, MA, USA). TGA/DSC and TMA were conducted using NETZSCH STA 449 F5 and TMA 402 F3 instruments (Selb, Germany), respectively. *D_k_* and *D_f_* were measured on a Keysight DPS 50 platform (Santa Rosa, CA, USA) according to the IPC-TM-650 [34] and ASTM D3380 standards. Tensile and elongation properties were obtained following the ASTM D412-C standard. Pull-off adhesion strength was measured with an SH-M adhesion tester (Hangzhou, China) following the ASTM D4541 standard.

### 3.2. Materials

5-Ethylidene-2-norbornene (ENB, chemically pure, Aladdin), phenol (chemically pure, Aladdin, Shanghai, China), epichlorohydrin (analytical grade, Aladdin), boron trifluoride diethyl etherate (chemically pure, Aladdin), cyanogen bromide (chemically pure, Aladdin), triethylamine (chemically pure, Sinopharm Chemical Reagent Co., Ltd., Shanghai, China), tetramethylammonium bromide (chemically pure, Macklin, Shanghai, China), toluene (analytical grade, Sinopharm Chemical Reagent Co., Ltd.), acetone (analytical grade, Sinopharm Chemical Reagent Co., Ltd.), sodium hydroxide (analytical grade, Sinopharm Chemical Reagent Co., Ltd.), anhydrous ethanol (analytical grade, Sinopharm Chemical Reagent Co., Ltd.), zinc powder (chemically pure, Sinopharm Chemical Reagent Co., Ltd.), sodium chloride (analytical grade, Sinopharm Chemical Reagent Co., Ltd.), anhydrous sodium carbonate (analytical grade, Aladdin), and commercial DGEBA epoxy resin (E-51, industrial grade, Oton Chemical Co., Ltd., Shanghai, China).

### 3.3. Synthesis of ENBNO

Typically, under a N_2_ atmosphere, 1.2 mol of phenol and 20 mL of toluene were introduced into a 250 mL three-necked flask and thoroughly mixed. The reaction system was heated to 70 °C, followed by the addition of 1 mL of boron trifluoride diethyl etherate (BF_3_·Et_2_O). The temperature was then increased to 75 °C, and 0.2 mol of ENB was slowly added dropwise over 1.5 h. After the addition was complete, the mixture was maintained at 75 °C with continuous stirring for an additional 1 h. Subsequently, 26.4 g of zinc powder was added as a decolorizing agent, and the temperature was raised to 140 °C for a further 3 h of reaction. Upon completion, the reaction mixture was cooled to room temperature and filtered to remove the zinc powder. The filtrate was washed three times with 0.5 wt% aqueous sodium carbonate solution followed by distilled water. Finally, excess toluene and phenol were removed by vacuum distillation, affording the ENBNO product a yield of ~90%.

### 3.4. Synthesis of ENBDE

Typically, to a three-necked flask, 0.156 mol of ENBNO, 117.3 mL of epichlorohydrin, and 0.03 mol of tetramethylammonium bromide were charged under continuous stirring to ensure a homogeneous mixture. The reaction system was then heated to 80 °C and maintained at this temperature for 2 h to facilitate the growth of polymer chains. Upon completion of this stage, the mixture was cooled to 70 °C, followed by the dropwise addition of 288 mL of a NaOH aqueous solution (48 wt%) over a period of 1 h. The reaction was further stirred at constant temperature for an additional 1 h to ensure completion. After the reaction, the system was allowed to cool naturally to room temperature. The organic phase was washed three times with distilled water to remove residual NaOH and other water-soluble impurities. Finally, excess epichlorohydrin was removed via vacuum distillation, yielding purified ENBDE with an approximate yield of ~83%.

### 3.5. Synthesis of ENBCY

In a typical process, under N_2_ protection, 0.236 mol of BrCN was added dropwise to 75 mL of acetone maintained at −25 °C under stirring for 30 min. Subsequently, 0.21 mol of triethylamine, 0.104 mol of ENBNO and 25 mL of acetone solution were slowly introduced into the solution, which was maintained at −15 °C during the addition. After the addition was completed, the reaction mixture was gradually increased to 0 °C and held for an additional 2 h. Then, the reaction mixture was filtered and washed with acetone three times. The filtrate was collected and re-dispersed in 100 mL of dichloromethane. This solution was then washed with 10% NaCl solution and distilled water to remove unreacted materials. The organic phase was subsequently dried using anhydrous sodium carbonate. Finally, the solvent was removed using an evaporator under vacuum to obtain the ENBCY with a yield of ~90%.

### 3.6. Detailed Test Conditions

FTIR tests were performed in transmission mode at a resolution of 4 cm^−1^. Samples were ground, mixed with dried KBr powder and pressed into pellets before measurement. Deuterated chloroform was used as the solvent for ^1^H-NMR characterization. Mechanical tests on fully cured resin specimens complied with China National Standard GB/T 2568—1995 [35], GB/T 41929—2022 [36] and GB/T 2411—2008 [37]. Cylindrical samples (4 mm–6 mm diameter, 5 mm–8 mm height) were used for TMA, and powder samples for TGA. All samples with different formulations were cured at 185 °C for 2 h. Moisture absorption tests followed GB/T 1034—2008 [38] and ISO 62:2008 [39]. Specimens were dried at 50 °C for 24 h, then immersed in distilled water at 23 °C ± 1 °C for 24 h.

Instrument and Configuration for dielectric properties: Measurements were performed using a Keysight (Agilent, Santa Clara, CA, USA) E5071C ENA Vector Network Analyzer (VNA) coupled with a precision rectangular waveguide test fixture (WR-90 band) designated for high-frequency sweeps. Sample Dimensions: The cured resin matrices were machined into smooth, precise rectangular specimens with standardized dimensions of 22.86 mm × 10.16 mm and a thickness of 2.0 mm–3.0 mm to match the internal dimensions of the waveguide cell perfectly, eliminating air-gap errors. The specialist performed this test after resizing our samples with precision grinding (the original size of our samples is 23 mm × 11 mm). Calibration protocol: A rigorous Thru-Reflect-Line (TRL) calibration sequence was implemented using a certified Keysight calibration kit prior to sample evaluation to eliminate systematic errors from the coaxial cables, adaptors, and fixtures. Measurement Uncertainty: The measurement software utilized the standard Nicolson–Ross–Weir (NRW) algorithm to extract intrinsic permittivity parameters. The instrument configuration yields a relative measurement uncertainty of ±0.05 for the *D_k_* and ±0.0005 for the *D_f_*, established via triplicate evaluations of standard reference materials.

### 3.7. Determination of Epoxy Value

Epoxy value refers to the amount of substance of epoxy groups contained per 100 g of resin. In this work, the hydrochloric acid-acetone method was adopted to determine the epoxy value in accordance with the China National Standard GB/T 1677-2023 [40] Determination of epoxy value for plasticizers. The epoxy value for ENBDE was measured to be 0.35 mol/100 g.

### 3.8. Determination of Viscosity

Viscosity was tested using a rotational viscometer at room temperature following relevant standards. The viscosities of ENBCY and ENBDE were 745.75 Pa·s and 7539 Pa·s, respectively.

### 3.9. Determination of Cyanate Conversion

The cyanate conversion of the transformation from ENBNO to ENBCY was determined by ^1^H NMR spectroscopy according to the consumption of hydroxyl proton signals [41]. Characteristic peaks of the unreactive molecular skeleton were adopted as the internal standard to normalize the signal intensity of hydroxyl groups. The conversion rate was calculated by comparing the normalized hydroxyl signal intensities of ENBCY product and ENBNO precursor, and the calculated conversion was approximately ~93%.

## 4. Conclusions

In summary, novel ENB-based epoxy and cyanate ester monomers were synthesized and blended with commercial DGEBA to construct high-performance thermosetting networks. The rigid, low-polarity norbornene structure effectively improves the thermal stability, heat resistance, high-frequency dielectric properties, moisture resistance and bonding performance of the modified resins. A typical performance trade-off exists in this system: the improved thermal and dielectric properties are accompanied by a slight reduction in tensile strength. Quantitatively, the optimized resin exhibits *D_k_* of 2.57 and *D_f_* of 0.0068 at 10 GHz, which is comparable to state-of-the-art low-k epoxy systems (*D_k_* = 2.1–2.7, *D_f_* = 0.003–0.008) [42] and far outperforms conventional DGEBA epoxies. This work also has certain limitations. The rigid NB structure weakens resin toughness, and the high cost of ENB-based monomers restricts large-scale industrial application. Nevertheless, this balanced epoxy/cyanate ester hybrid system is promising for high-frequency and high-speed electronic packaging.

## Figures and Tables

**Table 1:** Thermal properties of the cured epoxy resins ^a^.

ENBDE:DGEBA (Mass Ratio)	T_g_ (°C) ^b^	T_5%_ (°C) ^c^
100:0	249.2	351.5
75:25	204.3	332.6
50:50	174.9	319.3
25:75	116.6	293.5
0:100	95.4	247.6

^a^ Epoxy:ENBCY = 100:20 (mass ratio). ^b^ Determined by TMA test. ^c^ Determined by TGA test (T < T_g_).

**Table 2:** Mechanical properties of the cured epoxy resins ^a^.

ENBDE:DGEBA (Mass Ratio)	Hardness (Shore D)	Tensile Strength (MPa)	Tear Strength (N/mm)
100:0	71.6 ± 0.3	27.32 ± 0.42	35.10 ± 0.55
75:25	78.4 ± 0.2	30.43 ± 0.36	49.32 ± 0.42
50:50	80.9 ± 0.3	36.52 ± 0.27	55.43 ± 0.62
25:75	82.6 ± 0.4	43.14 ± 0.35	43.19 ± 0.39
0:100	77.2 ± 0.2	40.64 ± 0.38	38.10 ± 0.41

^a^ ASTM D412-C standard [22]. (Each group of samples was measured five times. The results are expressed as mean ± standard deviation).

**Table 3:** Dielectric, moisture adsorption and bonding properties of the cured epoxy resins.

ENBDE:DGEBA (Mass Ratio)	*D_k_*/*D_f_* ^a^	Moisture Absorption (%)	Bonding Strength (kN) ^b^
100:0	2.57/0.0068	0.68	0.21
75:25	2.62/0.0072	1.23	0.29
50:50	2.69/0.0079	1.66	0.26
25:75	3.02/0.012	1.79	0.19
0:100	3.33/0.036	2.42	0.23

^a^ ASTM D3380 standard [23]. ^b^ ASTM D4541 standard [24].

**Table 4:** Comparison of dielectric properties between the as-prepared resin system and reported epoxy resin systems in recent years.

Entry	Epoxy Resin System	*D_k_*/*D_f_*	Test Condition	Refs.
1	Dicyclopentadiene epoxy/3,5-Bis(benzoyloxy)benzyl-terminated poly(aryletherketone) with tetramethyl biphenyl groups	3.15/0.040	1 MHz	[25]
2	Benzoxazine-functionalized POSS/Dicyclopentadiene epoxy/Imidazole	2.21/0.0402	1 MHz	[26]
3	3-Trifluoromethylphenyl bisphenol epoxy/4-Trifluoromethylphenyl benzimidazole	3.38/0.02	10 MHz	[27]
4	3,3′,5,5′-Tetramethyl-4,4′-biphenyldiglycidyl ether/4,4′-Diaminodiphenylmethane	2.24/0.022	Not specified	[28]
5	1,3-Bis(4-(N,N-di(glycerol)amine)phenoxy)cyclododecane epoxy/Methylhexahydrophthalic anhydride	3.39/0.020	1 MHz	[29]
6	Dicyclopentadiene epoxy/Dicyclopentadiene-modified polyphenylene oxide	2.63/0.0104	1 MHz	[5]
7	E-51 bisphenol A epoxy/Methylhexahydrophthalic anhydride/Hyperbranched polyester with flexible chains	2.71/0.002	1 MHz	[30]
8	Tetramethyl biphenyl epoxy resin/Poly(aryl ether ketone) containing naphthalene and hydroxyl groups	1.90/0.010	1 MHz	[31]
9	Cresol novolac epoxy/Propargyl ether benzoxazine	2.62/0.027	1 GHz	[32]
10	ENBDE/DGEBA	2.69/0.0079	10 GHz	This work

## Data Availability

The data presented in this study are available on request from the corresponding author.

## References

[1] Watanabe A.O., Ali M., Sayeed S.Y.B., Tummala R.R., Pulugurtha M.R. (2021). A Review of 5G Front-End Systems Package Integration. IEEE Trans. Compon. Packag. Manuf. Technol..

[2] Zahidul Islam M.D., Fu Y., Deb H., Khalid Hasan M.D., Dong Y., Shi S. (2023). Polymer-based low dielectric constant and loss materials for high-speed communication network: Dielectric constants and challenges. Eur. Polym. J..

[3] Kuo C.-C., Lin Y.-C., Chen Y.-C., Wu P.-H., Ando S., Ueda M., Chen W.-C. (2021). Correlating the Molecular Structure of Polyimides with the Dielectric Constant and Dissipation Factor at a High Frequency of 10 GHz. ACS Appl. Polym. Mater..

[4] Zhang C., He X., Lu Q. (2024). High-frequency low-dielectric-loss in linear-backbone-structured polyimides with ester groups and ether bonds. Commun. Mater..

[5] Hwang H.-J., Hsu S.-W., Chung C.-L., Wang C.-S. (2008). Low dielectric epoxy resins from dicyclopentadiene-containing poly(phenylene oxide) novolac cured with dicyclopentadiene containing epoxy. React. Funct. Polym..

[6] Mohan P. (2013). A Critical Review: The Modification, Properties, and Applications of Epoxy Resins. Polym.-Plast. Technol. Eng..

[7] Jin F.-L., Li X., Park S.-J. (2015). Synthesis and application of epoxy resins: A review. J. Ind. Eng. Chem..

[8] Hwang H.-J., Li C.-H., Wang C.-S. (2006). Dielectric and thermal properties of dicyclopentadiene containing bismaleimide and cyanate ester. Part IV. Polymer.

[9] Shieh J.-Y., Hwang H.-J., Yang S.-P., Wang C.-S. (2005). Synthesis and properties of a cyanate ester containing dicyclopentadiene(II). J. Polym. Sci. Part A Polym. Chem..

[10] Li Y., Zhao P., Ju X., Jiang S., Zhang L., Li J., Li S., Li X., Zhang J. (2025). A fully polydicyclopentadiene skeletonized epoxy resin system and its fundamental properties as an electronic material. Chem. Commun..

[11] Commarieu B., Potier J., Compaore M., Dessureault S., Goodall B.L., Li X., Claverie J.P. (2016). Ultrahigh Tg Epoxy Thermosets Based on Insertion Polynorbornenes. Macromolecules.

[12] Commarieu B., Claverie J.P. (2015). Bypassing the lack of reactivity of endo-substituted norbornenes with the catalytic rectification–insertion mechanism. Chem. Sci..

[13] Bermeshev M.V., Bulgakov B.A., Genaev A.M., Kostina J.V., Bondarenko G.N., Finkelshtein E.S. (2014). Cationic Polymerization of Norbornene Derivatives in the Presence of Boranes. Macromolecules.

[14] Le Gac P.Y., Choqueuse D., Paris M., Recher G., Zimmer C., Melot D. (2013). Durability of polydicyclopentadiene under high temperature, high pressure and seawater (offshore oil production conditions). Polym. Degrad. Stab..

[15] Dimonie D., Dimonie M., Stoica S., Munteanu V., Abadie M.J. (2000). Some aspects of the thermal stability of linear polydicyclopentadiene (L-PDCPD). Polym. Degrad. Stab..

[16] Salunke A., Sasidharan S., Cherukattu Gopinathapanicker J., Kandasubramanian B., Anand A. (2021). Cyanate Ester—Epoxy Blends for Structural and Functional Composites. Ind. Eng. Chem. Res..

[17] Park S.-J., Heo G.-Y., Jin F.-L., Rhee K.Y. (2015). Effect of urethane functionality and number of epoxide groups on cure and mechanical behaviors of epoxy resins. Macromol. Res..

[18] Levita G., De Petris S., Marchetti A., Lazzeri A. (1991). Crosslink density and fracture toughness of epoxy resins. J. Mater. Sci..

[19] Puszka A., Podkościelna B. (2022). Special Issue: Synthesis, Processing, Structure and Properties of Polymer Materials. Polymers.

[20] Ederer J., Janoš P., Ecorchard P., Tolasz J., Štengl V., Beneš H., Perchacz M., Pop-Georgievski O. (2017). Determination of amino groups on functionalized graphene oxide for polyurethane nanomaterials: XPS quantitation vs. functional speciation. RSC Adv..

[21] Yang S., Zhang Q., Hu Y. (2016). Synthesis of a novel flame retardant containing phosphorus, nitrogen and boron and its application in flame-retardant epoxy resin. Polym. Degrad. Stab..

[22] (2016). Standard Test Methods for Vulcanized Rubber and Thermoplastic Elastomers—Tension (Die C).

[23] (2022). Standard Test Method for Relative Permittivity (Dielectric Constant) and Dissipation Factor of Polymer-Based Microwave Circuit Substrates.

[24] (2022). Standard Test Method for Pull-Off Strength of Coatings Using Portable Adhesion Testers.

[25] Meng H., Zhang Q., Lu M., Qu Z., Chen B., Xu C.-A., Lu M. (2021). Cure kinetics and properties of high-performance epoxy thermosets cured with active ester-terminated poly (aryl ether ketone). High Perform. Polym..

[26] Zhang S., Yan Y., Li X., Fan H., Ran Q., Fu Q., Gu Y. (2018). A novel ultra low-k nanocomposites of benzoxazinyl modified polyhedral oligomeric silsesquioxane and cyanate ester. Eur. Polym. J..

[27] Na T., Jiang H., Liu X., Zhao C. (2018). Preparation and properties of novel fluorinated epoxy resins cured with 4-trifluoromethyl phenylbenzimidazole for application in electronic materials. Eur. Polym. J..

[28] Guo H., Zheng J., Gan J., Liang L., Wu K., Lu M. (2015). Relationship between crosslinking structure and low dielectric constant of hydrophobic epoxies based on substituted biphenyl mesogenic units. RSC Adv..

[29] Kong L., Cheng Y., Jin Y., Qi T., Xiao F. (2016). Low k epoxy resin containing cycloaliphatic hydrocarbon with high crosslinking density. J. Appl. Polym. Sci..

[30] Wang Z., Zhang X., Weng L., Liu L. (2019). Low dielectric constant and high toughness epoxy resin based on hyperbranched polyester grafted by flexible chain modified. J. Mater. Sci. Mater. Electron..

[31] Zhang Y., Zhao C., Liu J., Na H. (2018). Preparation and characterization of ultralow dielectric and fibrous epoxy thermoset cured with poly(arylene ether ketone) containing phenolic hydroxyl groups. Eur. Polym. J..

[32] Lin C.H., Huang C.M., Wong T.I., Chang H.C., Juang T.Y., Su W.C. (2014). High-Tg and low-dielectric epoxy thermosets based on a propargyl ether-containing phosphinated benzoxazine. J. Polym. Sci. Part A Polym. Chem..

[33] Gunya A., Kúdelčík J., Hardoň Š., Janek M. (2025). Thermodielectric Properties of Polyurethane Composites with Aluminium Nitride and Wurtzite Boron Nitride Microfillers: Analysis Below and near Percolation Threshold. Sensors.

[34] (1998). Test Methods Manual.

[35] (1995). Test Method for Tensile Properties of Resin Casting Body.

[36] (2022). Plastics—Epoxy Resins—Test Methods.

[37] (2008). Plastics and Ebonite—Determination of Indentation Hardness by Means of a Durometer (Shore Hardness).

[38] (2008). Plastics—Determination of Water Absorption.

[39] (2008). Plastics—Determination of Water Absorption.

[40] (2023). Plasticizers—Determination of Epoxy Value.

[41] Galukhin A., Nosov R. (2019). Synthesis of Cyanate Esters Based on Mono-O-Methylated Bisphenols with Sulfur-Containing Bridges. Molecules.

[42] Wu Y., Fan X., Wang Z., Zhang Z., Liu Z. (2024). A mini-review of ultra-low dielectric constant intrinsic epoxy resins: Mechanism, preparation and application. Polym. Adv. Technol..

